# Dissecting human antibody responses: useful, basic and surprising findings

**DOI:** 10.15252/emmm.201808879

**Published:** 2018-01-23

**Authors:** Antonio Lanzavecchia

**Affiliations:** ^1^ Institute for Research in Biomedicine Università della Svizzera Italiana Bellinzona Switzerland

**Keywords:** Immunology, Microbiology, Virology & Host Pathogen Interaction

## Abstract

Human memory B cells and plasma cells represent a rich source of antibodies that have been selected in response to human pathogens. In the last decade, different methods have been developed to interrogate the human memory repertoire and isolate monoclonal antibodies. I will discuss how a target‐agnostic approach based on high‐throughput screening of antibodies produced by cultured B cells and plasma cells has not only provided potent and broadly neutralizing antibodies against a range of pathogens, but has also advanced our understanding of basic aspects of the immune response, from host–pathogen interaction to the role of somatic mutations in affinity maturation and in the diversification of the antibody response. Most surprisingly, this approach has also revealed a new mechanism of diversification based on templated insertion of non‐Ig DNA into antibody genes that we discovered in the context of the immune response to malaria infection.

## A trove of antibodies

Memory B cells represent the repository of the immune experience of an individual. They are selected in the germinal centres, where they undergo a process of somatic mutations and selection by antigen and T helper cells. Memory B cells persist for a life time and can rapidly respond to a booster immunization by generating large numbers of plasma cells that are transiently present in the blood and localize to surviving niches in the bone marrow as long‐lived plasma cells that are the main source of serum antibodies.

The interrogation of memory B cell and plasma cell repertoires of humans offers unique opportunities. As compared to mice, humans are naturally exposed to their own pathogens, often through recurrent infections, and are therefore a better source of antibodies that target the most relevant antigens and can reveal mechanisms of host–pathogen interaction. Furthermore, given the genetic variability of the human population and the idiosyncrasies of the immune response, some individuals may generate antibodies with unusual potency or breadth. Finally, studies of the antibody response in humans can shed light on basic aspects of the immune response, such as the role of somatic mutations and the mechanisms that underpin the generation of broadly neutralizing antibodies or autoantibodies, and possibly uncover examples of such mechanisms that may be uniquely developed or better identified in humans.

## A target‐agnostic approach to identify functional antibodies

**Figure 1:**
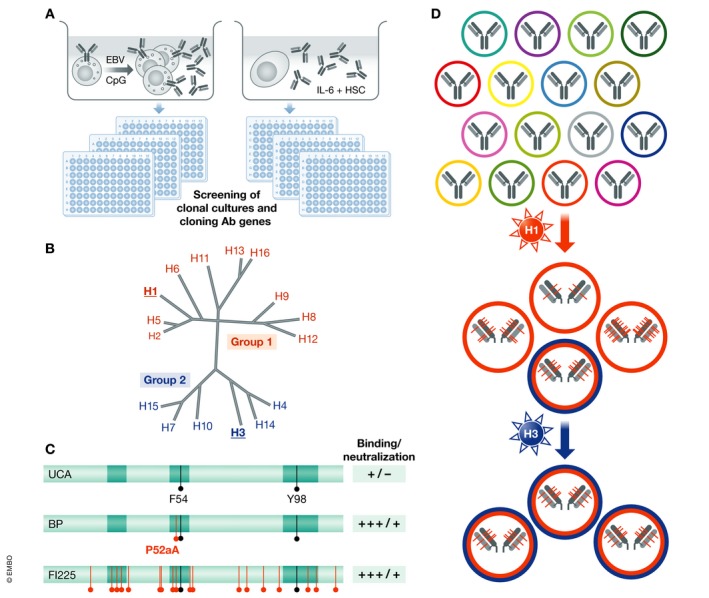
Dissecting human antibody responses to influenza virus: VH‐gene polymorphism and somatic mutations (A) Target‐agnostic approaches to interrogate human memory B cell or plasma cell repertoires based on high‐throughput screening of clonal cultures. (B) Protein distance of influenza A HA subtypes; H1 (group 1) and H3 (group 2) share only 35% amino acid identity. (C) Public antibodies that neutralize group 1 influenza viruses use VH1‐69 alleles with F54 and have a 13‐amino acid HCDR3 with Y98 (black lines). They can achieve high affinity through a single P52aA mutation (red). Shown are the functional properties of the UCA, of the first branch point (BP) and of a mature antibody (FI225) carrying several redundant mutations. (D) Schematic view of the developmental pathway of pan‐influenza neutralizing antibodies. A high‐affinity precursor specific for group 1 viruses (H1N1) generates, through somatic mutations, a variant that is expanded by stimulation with a group 2 virus (H3N2) in the absence of further mutations.

In the last decade, several methods have been developed to isolate human monoclonal antibodies. In principle, they come down to two basic approaches. The first approach consists of the isolation of single antigen‐specific B cells using a fluorescently tagged antigen as a bait, followed by cloning of immunoglobulin genes and expression of the recombinant antibodies in a cell line. This method is very effective, but requires that the target antigen is known and available in a purified form. In my laboratory, we developed an alternative approach based on high‐throughput screening of the antibodies produced by cultured B cells or plasma cells (Fig 1A). Memory B cells from selected donors are immortalized with high efficiency (>30%) by combining Epstein–Barr virus (that delivers signal 1 and signal 2) with a TLR7 or TLR9 agonist (that delivers signal 3 required for potent activation of memory B cells) (Traggiai *et al*, 2004). The immortalized B cells proliferate and secrete large amounts of antibodies in the culture supernatant. Antigen‐specific plasma cells recovered following a booster immunization have lost proliferative capacity, but can be kept alive in cultures supplemented with IL‐6 and stromal cells. In these cultures that mimic the survival niches of the bone marrow, a single plasma cell secretes antibodies at a constant rate of 100 pg/day (Corti *et al*, 2011). To facilitate the isolation of specific antibodies and the cloning of the VH/VL genes, both immortalized B cells and plasma cells are seeded in clonal conditions and cultures are handled with an automated liquid handling system. There are distinctive advantages in screening the repertoire of secreted antibodies. The first is that the approach is target‐agnostic, since screening can be done using functional assays, such as virus neutralization, without *a priori* knowledge of the target antigens, using even whole bacteria or parasites. Second, it is well suited to identify antibodies that cross‐react with different antigens, since parallel screenings can be performed against multiple targets. Third, it bypasses the need to sequence and express large numbers of antibody sequences since cells of interest are selected based on the initial screenings.

## Neutralizing antibodies for passive vaccination and vaccine design

A relevant example of the utility of the target‐agnostic approach comes from a study of the antibody response to human cytomegalovirus (HCMV), a complex herpesvirus expressing 20 different surface glycoproteins that causes serious pathology in the foetus and in immunocompromised patients. By screening for the capacity to neutralize the wild‐type virus, we isolated antibodies that were 1,000‐fold more potent than antibodies to the fusion protein gB and identified their viral target as a pentameric complex formed by gH, gL, pUL128, pUL130 and pUL131A. We then produced a soluble form of this pentamer and found that it can elicit, in mice, neutralizing antibody titres that exceeded by more than 100‐fold those induced in humans by natural infection (Kabanova *et al*, 2014). These studies illustrate a general approach of “analytic vaccinology” where the neutralizing antibodies are used for the identification of the target antigen and for the quality control of the recombinant vaccine.

The possibility of performing parallel screening against multiple targets was instrumental for the identification of broadly reactive antibodies capable of neutralizing multiple viruses. These include the antibodies FI6 and FY1, which bind to conserved epitopes in the stem region of influenza hemagglutinin (HA) and neutralize all influenza A viruses (Corti *et al*, 2011; Kallewaard *et al*, 2016), and MPE8, an antibody that binds to a unique conserved site on the pre‐fusion form of the F protein and neutralizes four different paramyxoviruses, including human RSV and MPV (Corti *et al*, 2013). These antibodies are promising candidates for prophylaxis and therapy of infections since they trigger effector functions and target conserved structures, thus limiting the possibility of selecting escape mutants. In addition, they represent useful tools for the design of stabilized pre‐fusion glycoprotein vaccines.

A straightforward application of the method lies in the rapid isolation of neutralizing antibodies against emerging pathogens, such as Ebola virus, SARS (Traggiai *et al*, 2004) and MERS coronaviruses. In the case of MERS, it was possible, in only 4 months, to go from a sample of memory B cells to preclinical validation of a neutralizing antibody and the generation of a cell line producing the antibody in grams/L amounts. Similar studies on Dengue and Zika viruses defined different classes of antibodies endowed with neutralizing or infection‐enhancing activity.

## V‐gene polymorphism and somatic mutations shaping antibody responses

The interrogation of the memory B‐cell repertoire offers the opportunity to investigate, in biologically and medically relevant settings, fundamental aspects of the human immune response, such as the relative contribution of germ line V‐gene polymorphism, VDJ junctional diversity and somatic mutations to antigenic specificity. An interesting example comes from the study of antibodies that neutralize group 1 influenza viruses and that are produced by most individuals following infection or vaccination. As reported by several laboratories, these “public” antibodies use VH1‐69 and bind, exclusively through the VH, to a conserved region in the HA stem. By isolating a large number of sister clones and by performing a genealogical analysis of different clonal families, we found that the unmutated common ancestors (UCA) have low affinity for group 1 HA and do not neutralize infection (Fig 1B and C). Remarkably, the first branch points acquire through a single P52aA mutation in HCDR2, high affinity and neutralizing activity, comparable to that of the antibodies isolated from memory B cells that carried up to 15–30 amino acid substitution (Pappas *et al*, 2014). These findings demonstrate that affinity maturation can be achieved rapidly, through a single mutation, and that numerous and redundant somatic mutations continue to accumulate, providing an extensive diversification within the proliferating clone. Interestingly, in these antibodies, a critical contact residue, F54, is germline‐encoded, but this position is dimorphic, with different alleles carrying either F or L. Consequently, individuals who lack a F54‐encoding allele cannot produce the public VH1‐69 antibody response, while individuals that have a F54 allele have a high frequency of precursors, with the only additional requirement being a 13‐amino acid HCDR3 with a Y98 that makes a critical contact site with the antigen. These findings underline the impact that VH‐gene polymorphism can have on the antibody response and suggest that certain alleles may play the role of immune response (Ir) genes and become positively selected in the population exposed to a given pathogen.

The genealogical analysis of the two pan‐influenza neutralizing antibodies, FI6 and FY1, showed that the corresponding UCAs have already high binding affinity and neutralizing activity, but only on group 1 viruses and acquire the capacity to neutralize group 2 viruses through somatic mutations (Kallewaard *et al*, 2016). These findings are consistent with a model where pan‐influenza neutralizing antibodies arise from the priming of high‐affinity precursors by a group 1 virus (in this case H1N1), followed by somatic mutations and generation of a variant with broader reactivity to group 2 viruses that can be boosted by a group 2 virus such as H3N2 (Fig 1D). Importantly, the isolation of cells producing the identical FI6 antibody for 4 consecutive years in response to vaccination or infection indicates that secondary antibody responses are to a large extent independent from germinal centres, a factor that allows the antibody to retain the original specificity. The genealogical analysis of the neutralizing antibody MPE8 revealed a similar mechanism, with the UCA possessing high affinity for RSV only and MPE8 acquiring cross‐reactivity to MPV through somatic mutations (Corti *et al*, 2013).

The extensive antibody diversification observed in humans can increase antibody affinity and promote breadth, but comes at the price of generating many new specificities that may cross‐react with self‐antigens. The notion that autoantibodies can be generated by somatic mutations is supported in humans by the finding that the UCAs of autoantibodies specific for desmoglein 3 or GM‐CSF found in patients with severe autoimmune diseases do not bind to the respective self‐antigens (Di Zenzo *et al*, 2012; Piccoli *et al*, 2015). Thus, it is tempting to speculate that these autoantibodies may have been generated by somatic mutations in B‐cell clones responsive to a foreign antigen in the absence of structural mimicry. Importantly, the mechanisms of deletion or anergy that restrain naïve autoreactive B cells may not apply to activated/memory B cells, which have different triggering requirement and can traffic to peripheral tissues.

## A surprising finding: antibodies generated by templated insertions

As mentioned above, the target‐agnostic approach is especially suited to dissect the antibody response to complex pathogens in the absence of a detailed knowledge of the target antigens. We were interested in identifying antibodies that could broadly recognize the variant surface antigens (VSAs) of *Plasmodium falciparum,* the parasite causing malaria, which are expressed on the surface of infected erythrocytes (IEs). VSAs mediate adhesion of IEs to endothelia and are targets of antibodies that control disease, but their high number (>200 genes), their extensive polymorphism and their clonal expression provide the pathogen with a powerful chameleon‐like escape strategy.

**Figure 2:**
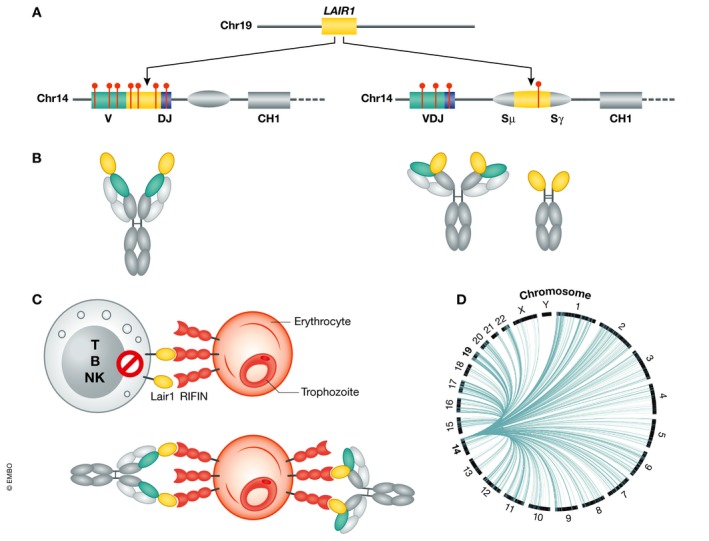
Antibody diversification by templated insertions (A) The *LAIR1* exon, flanked by short intronic sequences, is inserted in the V‐DJ junction or in the switch region. Somatic mutations (red lines) abolish collagen binding and increase binding to the malaria antigens RIFINs. (B) Schematic representation of the corresponding antibodies with the LAIR1 domain inserted at the tip of HCDR3 or at the VH‐CH1 elbow. (C) RIFINs bind to inhibitory receptors, including LAIR1, and represent a mechanism of parasite immune evasion. LAIR1‐containing antibodies bind to RIFINs on IEs, preventing their interaction with cellular LAIR1 and targeting IEs for destruction. (D) Templated inserts derived from transcribed genes encoded in different chromosomes are frequently found in the switch region of memory B cells in European blood donors (reproduced with permission from Pieper *et al* (2017)).

Out of a large Kenyan cohort, we initially selected two individuals with serum antibodies that cross‐agglutinated erythrocytes infected by different *P. falciparum* strains. From their memory B cells, we isolated a panel of broadly reactive monoclonal antibodies using staining of infected erythrocytes as a screening strategy. Surprisingly, all the broadly reactive antibodies had a unique structure, since they carried a large insert between the V and the DJ segments. The inserts, of approximately 400 bp, comprised the exon encoding the extracellular domain of the collagen‐binding inhibitory receptor LAIR1/CD305, encoded in the leucocyte receptor cluster on Chr. 19 (Tan *et al*, 2016). Given the insertion between V and DJ, the LAIR1 domain is positioned at the tip of the HCDR3 (Fig 2A and B). The target antigens of LAIR1‐containing antibodies were identified in some members of the RIFINs, a family of polymorphic VSAs expressed on the surface of IEs (Fig 2C). Importantly, the inserted LAIR1 domain was both necessary and sufficient for binding to RIFINs, as demonstrated by its insertion into an irrelevant antibody or by the production of LAIR1‐Ig fusion proteins.

To investigate how frequently LAIR1‐containing antibodies are produced, we screened two additional cohorts from Mali and Tanzania and found that up to 10% of the individuals have such antibodies in the serum (Pieper *et al*, 2017). This study led not only to the isolation of more LAIR1‐containing monoclonal antibodies, but also to the finding of a new insertion modality where the LAIR1 exon with 5′ and 3′ intronic sequences is inserted into the immunoglobulin switch region. This insertion leads, by exon shuffling, to the production of the original antibody and of a bispecific antibody containing the LAIR1 domain precisely placed at the elbow between the VH and CH1 domains (Fig 2A and B). In one case, the LAIR1 insertion in the switch region was accompanied by the genomic deletion of VH and CH1, generating a “camel‐like” LAIR1‐containing antibody.

In each individual analysed so far, the LAIR1‐containing antibodies dominate the response to IEs and are produced by a single B‐cell clone that contains a unique insert and undergoes extensive expansion and somatic mutations, thus demonstrating an extraordinary fitness. Somatic mutations in LAIR1 were more frequent when the insertion was at the V‐DJ junction, which is continually targeted by AID in germinal centres, and less frequent when the insertion was in the switch region, which is only transiently targeted by AID during switch recombination. Interestingly, somatic mutations in the LAIR1 domain eliminate collagen binding and modulate affinity and cross‐reactivity with different RIFINs. The finding that collagen binding was always lost, even when the somatic mutation mechanism was less effective, suggests that loss of self‐reactivity is a prerequisite for expansion of B cells making LAIR1‐containing antibodies.

The insertion of non‐VDJ sequences encoding structured domain into Ig genes is a new and potentially general concept. Our finding was facilitated by the huge expansion of LAIR1‐containing B cells and by the unique properties of the antibodies made. In an initial attempt to determine the generality of this phenomenon, we found frequent insertions in the switch region of memory B cells from European blood donors. Interestingly, the inserts largely originate from genes that are transcribed in B cells and are encoded in virtually all chromosomes and, in rare cases, comprised exons with frames compatible with expression (Fig 2D).

Malaria infection can cause chromosomal instability and translocations involving the switch region, suggesting that it may also play a causative role in the generation of templated insertions. However, the finding of frequent templated insertions in European blood donors is more consistent with a general mechanism that remains to be molecularly defined. The sites of insertion suggest a mechanism of patch‐repair of DNA double‐strand breaks induced by either RAG or AID during V‐DJ recombination or class switch. The integrity of the genomic LAIR1 loci in B‐cell clones producing LAIR1‐containing antibodies suggests a copy‐and‐paste mechanism. In view of the finding that the inserts contain intronic sequences and are derived from expressed genes, we favour the hypothesis that the templated DNA may originate from the resolution of stalled replication forks caused by a clash between transcription and DNA replication.

## Receptor‐based antibodies: emerging data for an old concept

Our findings suggest that the insertion of templated DNA represents a novel and general mechanism of antibody diversification that can provide a broad range of protein domains to be further diversified by somatic mutations. As exemplified by LAIR1, the insertion of a domain encoding a pathogen receptor can generate public antibodies that are effective against the pathogen. Distinctive advantages of these receptor‐based antibodies lie in the broad recognition of all pathogen variants, leaving no room for the selection of escape mutants, and in the possibility of diversifying the receptor domain through somatic mutations to increase binding to pathogen and decrease self‐reactivity. The new antibodies are reminiscent of Ehrlich's “side‐chain” theory of antibody production, where the antibody was essentially a secreted form of the receptor for the pathogen. We predict that other examples of receptor‐based antibodies will be found in response to a variety of human pathogens. Besides LAIR1, other candidates for the generation of receptor‐based antibodies to *P. falciparum* are ICAM1, an adhesion molecule that binds to IEs, and LILRB1, an inhibitory receptor that, like LAIR1, has been shown to bind to RIFINs. Similarly, insertions of ICAM1 or SLAM domains may generate receptor‐based antibodies that neutralize rhinoviruses or measles virus.

As a final remark, we realize that, by uncovering the ingenuity of Nature, we have learned new ways to engineer antibodies and, possibly, to edit antibodies in primary B cells using the endogenous AID activity. We also discovered how strong antibody responses can be generated by a single B‐cell clone, raising a case for adoptive B‐cell therapy.

## Conflict of interest

AL is scientific founder of Humabs BioMed, has substantial interest in antibodies developed by Humabs and is a shareholder of VIR Bio.
